# Predicting outcomes after blunt chest wall trauma: development and external validation of a new prognostic model

**DOI:** 10.1186/cc13254

**Published:** 2014-03-17

**Authors:** C Battle, S Lovett, H Hutchings, PA Evans

**Affiliations:** 1 Morriston Hospital, ABMU Health Board, Swansea, UK; 2 Swansea University, Swansea, UK

## Introduction

Blunt chest wall trauma accounts for over 15% of all trauma admissions to emergency departments worldwide [1]. Reported mortality rates vary between 4 and 60% [2]. Management of this patient group is challenging as a result of the delayed onset of complications. The aim of this study was to develop and validate a prognostic model that can be used to assist in the management of blunt chest wall trauma.

## Methods

There were two distinct phases to the overall study; the development and the validation phases. In the first study phase, the prognostic model was developed through the retrospective analysis of all blunt chest wall trauma patients (*n *= 274) presenting to the emergency department of a regional trauma centre in Wales (2009 to 2011). Multivariable logistic regression was used to develop the model and identify the significant predictors for the development of complications. The model's accuracy and predictive capabilities were assessed. In the second study phase, external validation of the model was completed in a multicentre prospective study (*n *= 237) in 2012. The model's accuracy and predictive capabilities were re-assessed for the validation sample. A risk score was developed for use in the clinical setting.

## Results

Significant predictors of the development of complications were age, number of rib fractures, chronic lung disease, use of preinjury anticoagulants and oxygen saturation levels. The final model demonstrated an excellent c-index of 0.97.

## Conclusion

In our two-phase study, we have developed and validated a prognostic model that can be used to assist in the management of blunt chest wall trauma patients. The final risk score provides the clinician with the probability of the development of complications for each individual patient.
